# Inhibition of mitochondrial glutaminase activity reverses acquired erlotinib resistance in non-small cell lung cancer

**DOI:** 10.18632/oncotarget.6311

**Published:** 2015-11-13

**Authors:** Caifeng Xie, Jiangbo Jin, Xujie Bao, Wei-Hua Zhan, Tian-Yu Han, Mingxi Gan, Chengfu Zhang, Jianbin Wang

**Affiliations:** ^1^ Institute of Translational Medicine, Nanchang University, Nanchang, Jiangxi, P. R. China

**Keywords:** non-small cell lung cancer, erlotinib, glutaminase inhibitor-968, epidermal growth factor receptor

## Abstract

The epidermal growth factor receptor (EGFR) tyrosine kinase inhibitor (TKI) erlotinib has been approved based on the clinical benefit in non-small cell lung cancer (NSCLC) patients over the past decade. Unfortunately, cancer cells become resistant to this agent *via* various mechanisms, and this limits the improvement in patient outcomes. Thus, it is urgent to develop novel agents to overcome erlotinib resistance. Here, we propose a novel strategy to overcome acquired erlotinib resistance in NSCLC by inhibiting glutaminase activity. Compound 968, an inhibitor of the glutaminase C (GAC), when combined with erlotinib potently inhibited the cell proliferation of erlotinib-resistant NSCLC cells HCC827ER and NCI-H1975. The combination of compound 968 and erlotinib not only decreased GAC and EGFR protein expression but also inhibited GAC activity in HCC827ER cells. The growth of erlotinib-resistant cells was glutamine-dependent as proved by GAC gene knocked down and rescue experiment. More importantly, compound 968 combined with erlotinib down-regulated the glutamine and glycolysis metabolism in erlotinib-resistant cells. Taken together, our study provides a valuable approach to overcome acquired erlotinib resistance by blocking glutamine metabolism and suggests that combination of EGFR-TKI and GAC inhibitor maybe a potential treatment strategy for acquired erlotinib-resistant NSCLC.

## INTRODUCTION

Lung cancer is the leading cause of cancer-related mortality worldwide, accounting for nearly 1.4–1.6 million deaths in each year [1–3]. In 2014, the overall estimation of 224,210 new cases is diagnosed, and 159,260 cases are estimated to finally die of lung cancer, accounting for 27.2% of all the cancer-related death in the United States [4]. In many countries, the mortality related to lung cancer continues to rise. The two main classification of lung cancer based on tumor histology are small cell lung carcinoma (SCLC) and non-small-cell lung carcinoma (NSCLC) [5]. Non-small cell lung cancer, which mainly includes squamous cell carcinoma, large cell carcinoma, and adenocarcinoma, accounts for nearly 85% of all cases of lung cancer [6].

Approximately 20% lung cancer patients occupy EGFR mutations that promote cancer cell growth [7]. This makes EGFR an important therapeutic target for the treatment of NSCLC. EGFR-TKIs, such as gefitinib and erlotinib, were reported to have therapeutic effects against NSCLC with EGFR activating mutations [8–10]. However, almost all tumors acquire resistance to EGFR-TKIs after varying periods of treatment time [11, 12]. Although the occurrence of genetic alterations such as T790M second mutation, *MET* amplification, hepatocyte growth factor (HGF) overexpression have been implicated [13–16], the precise mechanisms responsible for the acquired resistance to EGFR-TKIs still not well understood.

Malignant tumor cells exhibit considerably different metabolic requirements involved in glycolysis and glutamine metabolism compared to adjacent normal cells [17–19]. The earliest and best-known cancer metabolic anomaly is Warburg effect characterized by increased glycolysis and lactate production regardless of oxygen availability [20]. Therefore, targeting the peculiar metabolic pathways in cancer might be an effective strategy for cancer therapy. Recently, it was reported that enhanced glutamine metabolism as well as the expression of GLS occurred in both patients and cell lines resistant to EGFR-TKIs [21, 22]. Therefore, inhibition of glutamine metabolism may be a potential strategy against NSCLC.

Glutamine is the most abundant and versatile nutrient that plays a vital role in multiple metabolic processes and signaling in human cells. For glutamine metabolism, GLS is the key enzyme in the conversion of glutamine to glutamate and is expressed in many tissue cells and cancer cells [23–25]. GLS has two isoforms in human cells: GLS1 (known as kidney glutaminase) and GLS2 (known as liver glutaminase). GLS1 is a phosphate-activated enzyme with two major splice variants: a long form (KGA) and a short form (GAC) [26]. It was reported that GAC knocking-down resulted in more cell growth reduction than KGA knocking-down in several lung cancer cell lines indicating that GAC is the more essential GLS1 splice variant in NSCLC [27].

In our pervious study, we found a novel GAC inhibitor, named 968 (5-(3-bromo-4-(dimethylamino)phenyl)-2,2-dimethyl-2,3,5,6-tetrahydrobenzo[α]- phenanthridin-4(1H)-one). It blocked breast cancer cell proliferation, migration, invasion and the growth of tumors in mouse xenograft model but no inhibitory effects on normal cells [28]. Therefore, in the present study, we try to explore whether compound 968 can overcome the resistance to erlotinib in NSCLC by blocking glutamine metabolism, and to identify the effects of combined therapy of compound 968 and erlotinib on NSCLC.

## RESULTS

### The effects of erlotinib on human NSCLC-HCC827 and HCC827ER cells

**Figure 1:**
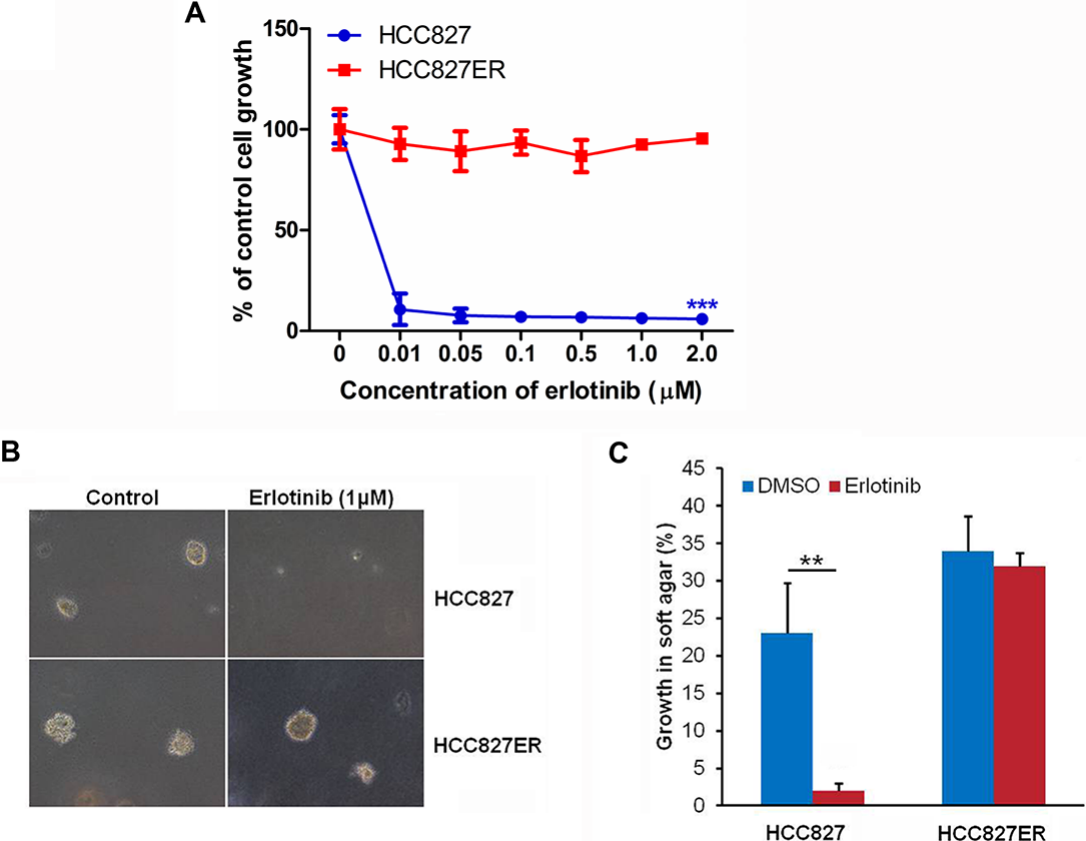
HCC827ER cells are resistant to erlotinib (**A**) Cell growth assay. HCC827 and HCC827ER cells were cultured in RPMI 1640 supplemented with 10% FBS, and were either treated with increasing concentrations of erlotinib for 48 h, or untreated. Cell growth was determined by crystal violet staining. Data represent the average of three independent experiments (mean ± SD). ***P < 0.001. (**B**) Soft agar assays. HCC827 and HCC827ER cells were mixed with RPMI 1640 supplemented with 0.3% agrose and 10% FBS and plated on top of RPMI 1640 supplemented with 0.5% agrose and 10% FBS. Cells were treated with erlotinib (1 μM), or untreated. Colonies were scored after 14 days of growth. 100% represents 500 cells counted. (**C**) Statistical analysis of colony formation in soft agar assays described above. The data represent the average of three independent experiments (mean ± SD). **P < 0.01.

Human NSCLC cell lines HCC827 (*EGFR* exon 19 deletion [delE746-A750]) and erlotinib-resistant HCC827ER cells harboring *MET* gene amplification were used in this study. We first confirmed the resistance of HCC827ER cells to erlotinib. As shown in Figure 1A, the growth of HCC827ER cells was not inhibited by erlotinib even at the concentration up to 2 μmol/L. However, HCC827 cells were unable to grow under these conditions, only 10% of parental HCC827 cells survived after exposure to 10 nmol/L erlotinib (***P < 0.001).

To investigate the anchorage independent growth of malignant cells, the soft agar assay was performed. For HCC827ER cells, they formed big colonies whether treated with or without 1 μM erlotinib. However, for HCC827 cells, they formed colonies, but colonies disappeared after treatment with 1 μM erlotinib (**P < 0.01, Figure 1B and 1C). These results further confirm that HCC827ER cells were resistant to erlotinib.

### The growth of HCC827 and HCC827ER cells depends on glutamine availability

**Figure 2:**
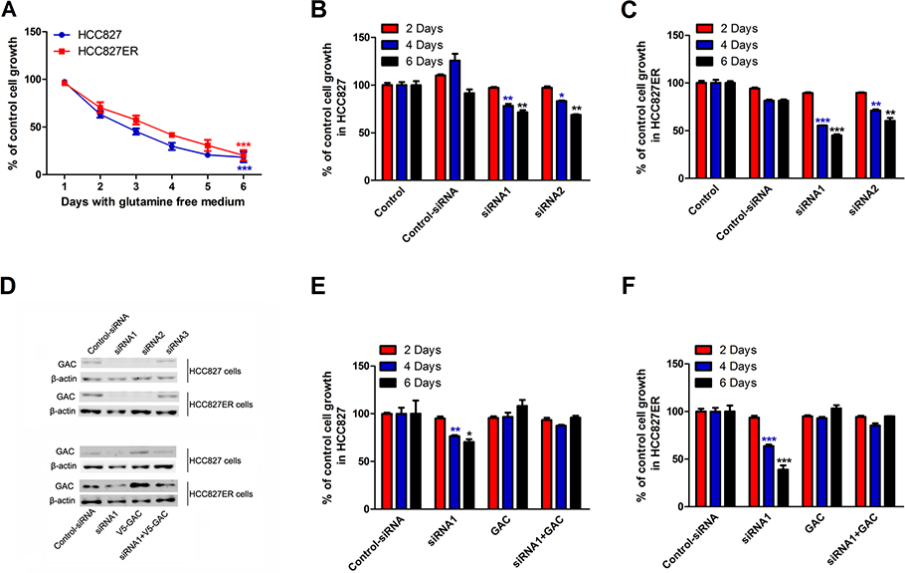
The growth of HCC827 and HCC827ER cells depends on glutamine (**A**) HCC827 (1 × 10^5^ cells per well) and HCC827ER cells (1 × 10^5^ cells per well) were cultured in RPMI 1640 medium with glutamine or without for 6 days. The cell numbers were counted every day. The number of cells cultured in medium with glutamine (300 mg/L L-Glutamine) was calculated as control. Data are shown as means ± S.D. of three experiments. ***P < 0.001. (**B**) HCC827 cells were transfected with control siRNA or siRNA1 and siRNA2 targeting GAC. Cells were grown for the indicated number days and counted. Untreated cells were used as control. Data represent the average of three independent experiments (mean ± SD). *P < 0.05, **P < 0.01. (**C**) HCC827ER cells were transfected with control siRNA or siRNA1 and siRNA2 targeting GAC. Cells were grown for the indicated number days and counted. Untreated cells were used as control. Data represent the average of three independent experiments (mean ± SD). **P < 0.01, ***P < 0.001. (**D**) The efficiencies of the siRNA knocking down of GAC in both HCC827 and HCC827ER cells were determined by Western blot (top panel). The rescue efficiencies of GAC in HCC827 and HCC827ER cells were determined after the transfection of pCDNA 3.1-V5-GAC (bottom panel). (**E**) HCC827 cells were transfected with control siRNA or siRNA1 and siRNA2 targeting GAC. After 24 hours, V5-GAC plasmid was transfected into HCC827 cells. The cell numbers were counted on the indicated days. Data represent the average of three independent experiments (mean ± SD). *P < 0.05, **P < 0.01. (**F**) HCC827ER cells were transfected with control siRNA or siRNA1 and siRNA2 targeting GAC. After 24 hours, V5-GAC plasmid was transfected into HCC827ER cells. The cell numbers were counted on the indicated days. Data represent the average of three independent experiments (mean ± SD). ***P < 0.001.

Some cancer cells use glutamine (Gln) to support anabolic processes that fuels their proliferation [29]. To evaluate the effects of glutamine metabolism in HCC827 and HCC827ER cells, we detected cell growth in the medium with or without glutamine. HCC827 and HCC827ER cells were incubated in glutamine free RPMI 1640 medium, and cell numbers were counted from 1 to 6 days. The cell number of HCC827 decreased from approximately 100% on day 1 to 18% on day 6 (Figure 2A, ***P < 0.001), and the similar results were observed for HCC827ER cells. These results indicate that the growth of both cells has great dependency on glutamine.

To further confirm the glutamine-dependency for both cell lines, we knocked down GAC by siRNAs in HCC827 and HCC827ER cells, and performed cell proliferation assays. As shown in Figure 2B and 2C, knocking down GAC by siRNA1 and siRNA2 significantly suppressed cell growth for both HCC827 (Figure 2B, *P < 0.05, **P < 0.01) and HCC827ER (Figure 2C, **P < 0.01 and ***P < 0.001) on day 4 and 6. The expressions of GAC was showed in Figure 2D. To further prove the important role of GAC played in the regulation of acquired resistance, we knocked down GAC, and then transfected GAC plasmid into the cells for a rescue experiment. The rescue efficiency in HCC827ER was much higher than that in HCC827 cells (Figure 2E and 2F).

All these results indicate that the growth of HCC827 and HCC827ER cells is glutamine dependent, and GAC plays a central role in regulating cell proliferation in erlotinib-resistant NSCLC cells.

### Combination of compound 968 and erlotinib has synergized inhibitory effects on the growth of HCC827ER cells

**Figure 3:**
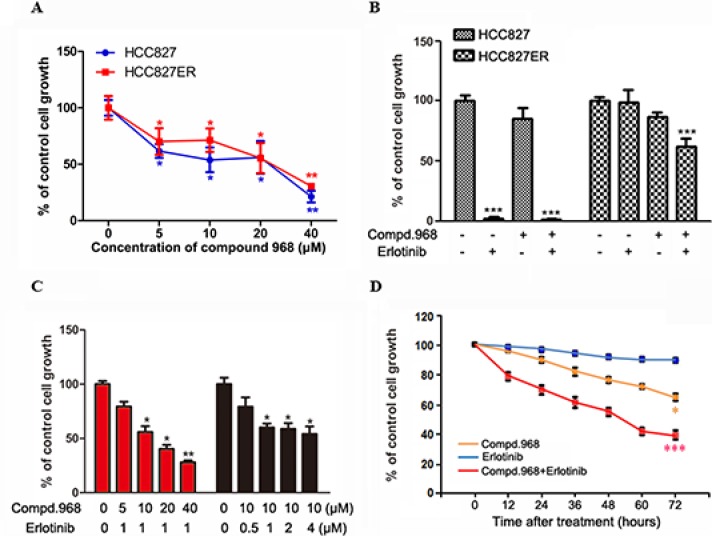
Combination of compound 968 and erlotinib has synergized inhibitory effects on the growth of HCC827ER cells (**A**) Saturation density assay. Cells were treated with or without increasing concentrations of compound 968 for 6 days, trypsinized and counted. The data represent the average of three independent experiments (mean ± SD). *P < 0.05, **P < 0.01. (**B**) HCC827 and HCC827ER cells were cultured in RPMI 1640 supplemented with 10% FBS, and were treated with compound 968 (10 μM), or erlotinib (1 μM), or compound 968 (10 μM) combined with erlotinib (1 μM) for 48 hours or untreated. Cell growth was determined by crystal violet staining. Data represent the average of three independent experiments (mean ± SD). ***P < 0.001. (**C**) HCC827ER cells were cultured in RPMI 1640 supplemented with 10% FBS, and were treated with erlotinib (1 μM) combined with increasing concentrations of compound 968 (5, 10, 20, and 40 μM), or treated with compound 968 (10 μM) combined with increasing concentrations of erlotinib (0.5, 1, 2, and 4 μM) for 48 hours or untreated. Cell growth was determined by crystal violet staining. Data represent the average of three independent experiments (mean ± SD). *P < 0.05, **P < 0.01. (**D**) Time course experiment. HCC827ER cells were cultured in RPMI 1640 supplemented with 10% FBS, and were treated with compound 968 (10 μM), or erlotinib (1 μM), or compound 968 (10 μM) combined with erlotinib (1 μM) for different time or untreated. Cell growth was determined by crystal violet staining. Data represent the average of three independent experiments (mean ± SD). *P < 0.05, ***P < 0.001.

Glutaminolysis and glutaminase (GLS) have been showed to be indispensable for cancer development and progression. Glutaminase C (GAC), a splice variant of kidney-type glutaminase, has been demonstrated to be the major form of GLS in tumor cells [22, 24]. In our previous study, we found a novel GAC inhibitor-compound 968 and proved that compound 968 had a potent inhibitory effect on the growth of breast cancer cells [23]. In present study, we already proved that the growth of HCC827 and HCC827ER cells is dependent on glutamine. We were wondering whether compound 968 had effects on the growth of HCC827 and HCC827ER cells. As shown in Figure 3A, compound 968 treatment at different concentrations significantly inhibited the cell growth of both HCC827 and HCC827ER cells (*P < 0.05, **P < 0.01) in saturation density assay. There was no significant difference statistically between HCC827 and HCC827ER cells for the inhibitory effects of compound 968, suggesting that compound 968 suppressed NSCLC cell proliferation through a pathway distinguished from erlotinib.

The effects of compound 968 combined with erlotinib on the growth of HCC827 and HCC827ER cells were further investigated by cell growth assay. The cell numbers for both HCC827 and HCC827ER cells decreased by 20% comparing with the control cells when treated with compound 968 for 48 hours. However, the growth of HCC827ER cells was inhibited potently when treated with the combination of compound 968 and erlotinib (Figure 3B, ***P < 0.001) indicating that compound 968 not only overcomes acquired erlotinib resistance but also restore sensitivity of HCC827ER cells to erlotinib.

To further investigate the mode of action for compound 968 combined with erlotinib on the growth of HCC827ER cells, we did cell growth assay by changing the concentration of one regent with the concentration of another one fixed. As shown in Figure 3C, HCC827ER cells were treated with increasing concentrations of compound 968 (5, 10, 20, and 40 μM) combined with 1 μM erlotinib for 48 hours. The cell growth decreased in a compound 968 dose dependent manner, and the cell number was reduced by 72% compared to untreated control cells at the concentration of 40 μM (Figure 3C, *P < 0.05, **P < 0.01). However, when HCC827ER cells were treated with increasing concentrations of erlotinib (0.5, 1.0, 2.0, 4.0 μM) combined with 10 μM compound 968, the growth of HCC827ER cells did not decrease in an erlotinib dose dependent manner (*P < 0.05).

Time-course experiments were also performed. HCC827ER cells were treated with erlotinib, compound 968, or compound 968 combined with erlotinib for 0, 12, 24, 36, 48, 60, and 72 hours respectively. Treatment with either erlotinib (1 μM) or compound 968 (10 μM) alone slightly or modestly suppressed the growth of HCC827ER cells. However, the combination of compound 968 (10 μM) and erlotinib (1 μM) potently inhibited the growth of HCC827ER cells in a time-dependent manner (Figure 3D). The cell numbers for combined group decreased from 100% to 38% compared to the control group (***P < 0.001).

In order to test whether compound 968 sensitized cancer cells to other anticancer drugs except erlotinib, gefitinib (the first selective inhibitor of EGFR) and cispatin (the first member of a class of platinum-containing anti-cancer drugs) were used for the evaluation. As shown in the Figure S1, compound 968 sensitized cancer cells to gefitinib at a high concentration, and the sensitization efficiency was lower than that to erlotinib. Furthermore, compound 968 had a weak effect on the cell sensitization to cispatin.

These findings suggest that compound 968 plays critical role in reversing acquired erlotinib and Gefitinib resistance for HCC827ER cells.

### Combination of compound 968 and erlotinib also decreases the growth of other NSCLC cell lines

**Figure 4:**
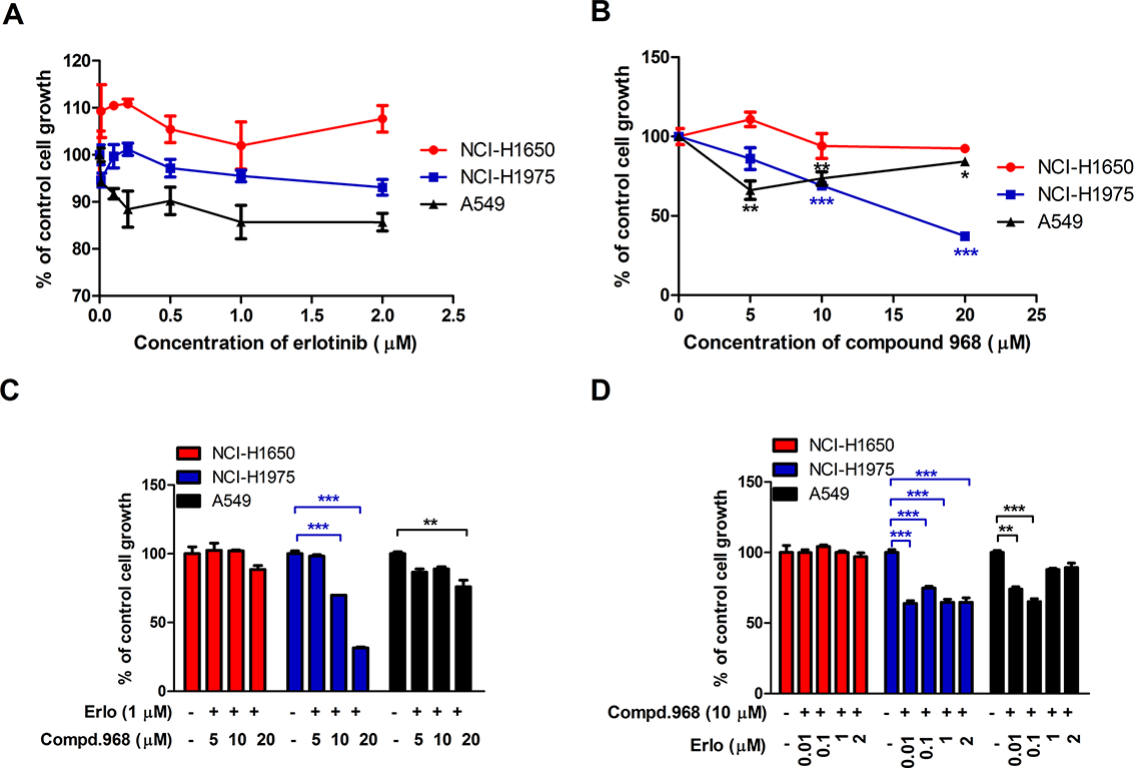
Combination of compound 968 and erlotinib also decreases the growth of other NSCLC cell lines (**A**) NCI-H1650, NCI-H1975 and A549 cells were cultured in RPMI 1640 supplemented with 10% FBS, and were treated with different concentrations of erlotinib (0.01, 0.05, 0.1, 0.5, 1, and 2 μM) for 48 h or untreated. Cell growth was determined by crystal violet staining. Data represent the average of three independent experiments (mean ± SD). (**B**) NCI-H1650, NCI-H1975 and A549 cells were cultured in RPMI 1640 supplemented with 10% FBS, and were treated with different concentrations of compound 968 (0, 5, 10, and 20 μM) for 48 h or untreated. Cell growth was determined by crystal violet staining. Data represent the average of three independent experiments (mean ± SD). *P < 0.05, **P < 0.01, ***P < 0.001. (**C**) NCI-H1650, NCI-H1975 and A549 cells were cultured in RPMI 1640 supplemented with 10% FBS, and were treated with erlotinib (1 μM) combined with increasing concentrations of compound 968 (5, 10, and 20 μM). Cell growth was determined by crystal violet staining. Data represent the average of three independent experiments (mean ± SD). **P < 0.01, ***P < 0.001. (**D**) NCI-H1650, NCI-H1975 and A549 cells were cultured in RPMI 1640 supplemented with 10% FBS, and were treated with compound 968 (10 μM) combined with increasing concentrations of erlotinib (0.01, 0.1, 1, and 2 μM) for 48 hours or untreated. Cell growth was determined by crystal violet staining. Data represent the average of three independent experiments (mean ± SD). **P < 0.01, ***P < 0.001.

To investigate whether similar phenomenon can be observed for other human NSCLC cell lines, three new cell lines (A549, NCI-H1975, and NCI-H1650) were used for cell proliferation assay. Among them, A549 cells were EGFR wild type, NCI-H1975 and NCI-H1650 cells were EGFR mutants with the same kinase domain mutation (L858R and ΔE746-A750). They also have additional changes such as T790M in NCI-H1975 and PTEN loss in NCI-H1650 [30, 31]. Erlotinib did not inhibit significantly the growth of all these cell lines even at the concentration of 2 μM indicating that all these cell lines were insensitive to erlotinib (Figure 4A). However, compound 968 suppressed the cell proliferation of A549 and NCI-H1975 cells but had no effects on the growth of NCI-H1650 cells (Figure 4B). Furthermore, compound 968 showed much stronger inhibitory effect on NCI-H1975 than A549 cells.

The effects of erlotinib combined with compound 968 on the three NSCLC cell lines were also investigate. As shown in Figure 4C, NCI-H1650, NCI-H1975 and A549 cells were treated with increasing concentrations of compound 968 (5, 10, and 20 μM) combined with 1 μM erlotinib for 48 hours. The growth of NCI-H1975 cells decreased in a compound 968 dose dependent manner, and the cell number was decreased by 70% compared to untreated control cells at the concentration of 20 μM (Figure 4C, ***P < 0.001). The growth of A549 cells decreased moderately at the concentration of 20 μM. However, the growth of NCI-H1650 cells did not decrease significantly. When those cell lines were treated with increasing concentrations of erlotinib (0.01, 0.1, 1.0, and 2.0 μM) combined with 10 μM compound 968, the growth of NCI-H1975 and A549 cells did not decrease in an erlotinib dose dependent manner (Figure 4D), while it was no effects on the growth of NCI-H1650 cells under such condition.

T790M mutation in EGFR is detectable in approximately 50% of patients with NSCLC who relapse after an initial response to TKIs. It is regarded as a marker for acquired resistance to EGFR-TKIs. Our results suggest that compound 968 combined with erlotinib can overcome the acquired resistance to erlotinib in NSCLC especially with EGFR (T790M) mutation.

### Compound 968 reverses acquired erlotinib resistance by blocking glutaminase activity in HCC827ER cells

**Figure 5:**
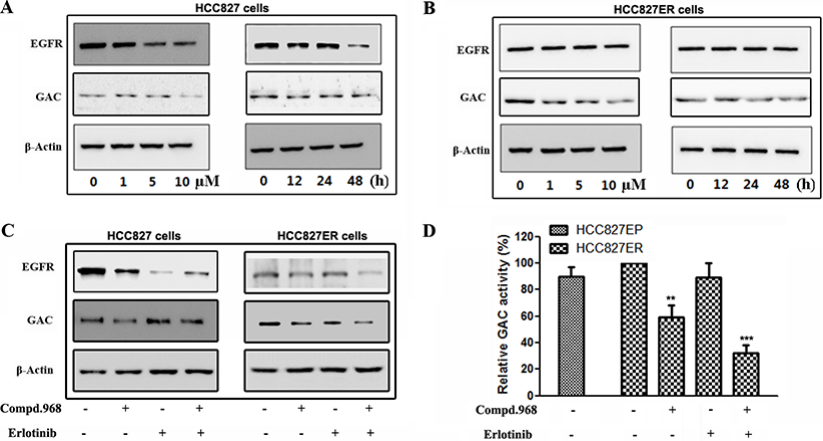
Compound 968 reverses acquired erlotinib resistance by blocking GAC activity in HCC827ER cells (**A**) HCC827 cells were cultured in RPMI 1640 supplemented with 10% FBS, and were treated with compound 968 at 0, 1, 5, and 10 μM concentrations for 48 hours (left panel) or treated with 10 μM compound 968 for 0, 12, 24 and 48 hours (right panel). The EGFR and GAC expressions were determined by Western blot. (**B**) HCC827ER cells were cultured in RPMI 1640 supplemented with 10% FBS, and were treated with compound 968 at 0, 1, 5, and 10 μM concentrations for 48 hours (left panel) or treated with 10 μM compound 968 for 0, 12, 24 and 48 hours (right panel). The EGFR and GAC expressions were deterimine by Western blot. (**C**) HCC827 (left panel) and HCC827ER (right panel) cells were cultured in RPMI 1640 supplemented with 10% FBS, and were treated with compound 968 (10 μM), or erlotinib (1 μM), or compound 968 (10 μM) combined with erlotinib (1 μM) for 48 hours respectively. The EGFR and GAC expressions were determined by Western blot. (**D**) HCC827 and HCC827ER cells were cultured in RPMI 1640 supplemented with 10% FBS, and were treated with compound 968 (10 μM), or erlotinib (1 μM), or compound 968 (10 μM) combined with erlotinib (1 μM) for 48 hours respectively, then mitochondria was isolated from different cells, and GAC activity was determined by glutaminase activity assay. Data represent the average of three independent experiments (mean ± SD). **P < 0.01, ***P < 0.001.

EGFR is the target of erlotinib, and we are wondering whether compound 968 overcomes acquired erlotinib resistance by inhibiting EGFR. We investigated the expressions of EGFR and GAC in both HCC827 and HCC827ER cells with compound 968 treatment. Compound 968 decreased the expression of EGFR and GAC in HCC827 cells in a dose- and a time-dependent manner (Figure 5A). Intriguingly, although the expression of GAC was inhibited by compound 968 in HCC827ER, the EGFR expression in HCC827ER cells was not decreased by compound 968 treatment even at the highest concentration (10 μM) and with longest treatment time (48 h) (Figure 5B). We further evaluated the effects of compound 968 combined with erlotinib on EGFR and GAC expressions in both HCC827 and HCC827ER cells. The expression level of EGFR in HCC827 cells (Figure 5C, left panel) was higher than that in HCC827ER cells (Figure 5C, right panel), while the expression level of GAC was reversed. Although the expression of EGFR in HCC827ER cells was not significantly suppressed by compound 968 or erlotinib treatment alone, it was inhibited obviously by the combination of compound 968 and erlotinib (Figure 5C, right panel). This combination also decreased the expression level of GAC in HCC827ER cells. Statistical analysis of band intensities was showed in Figure S2.

To understand the molecular mechanism that compound 968 reverses acquired erlotinib resistance in HCC827ER cells, we tested the enzyme activity of GAC in human NSCLC cells (Figure 5D). The GAC activity in HCC827ER cells was slightly higher than that in HCC827 cells. Compound 968 treatment alone decreased the GAC activity of HCC827ER cells by about 40% compared with untreated cells, however, combination of compound 968 and erlotinib inhibited the GAC activity by approximately 70% compared with untreated cell (**P < 0.01, ***P < 0.001). All these results indicate that the combinatorial treatment potently reverses the acquired erlotinib resistance by suppressing the expressions of EGFR and GAC, and further GAC activity.

### Combination of compound 968 and erlotinib decreases glutamine and glycolysis metabolism in HCC827ER cells

**Figure 6:**
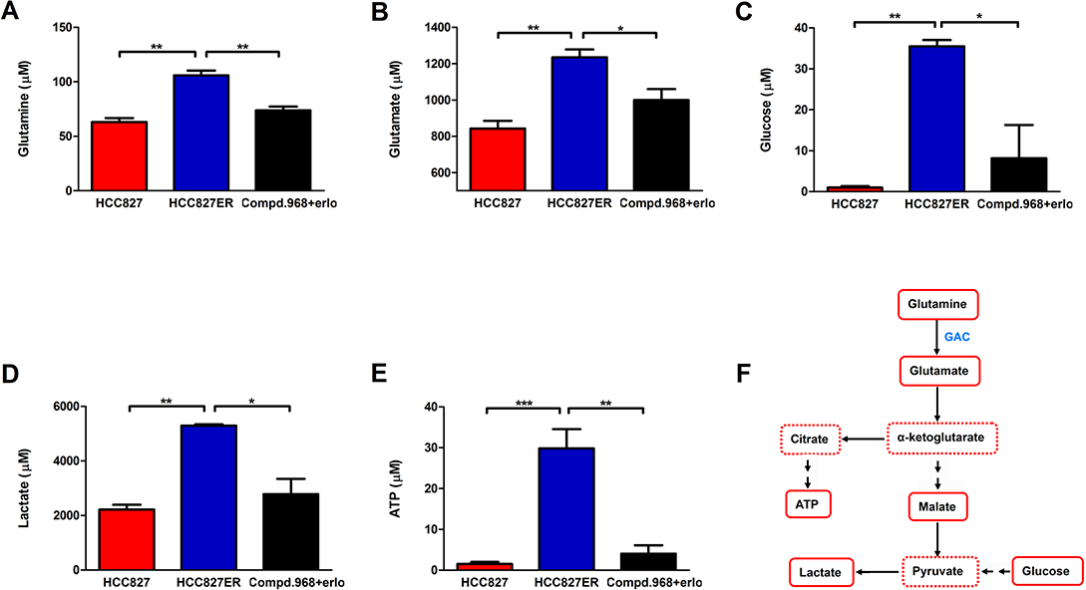
Combination of compound 968 and erlotinib inhibits the glutamine and glycolysis metabolism The concentrations of Glutamine (**A**), glutamate (**B**), glucose (**C**), lactate (**D**), and ATP (**E**) were detected in HCC827, HCC827ER cells or HCC827ER cells treated with compound 968 (10 μM) combined with erlotinib (1 μM). Data represent the average of three independent experiments (mean ± SD). *P < 0.05, **P < 0.01, ***P < 0.001. (**F**) A summary map of metabolites from glycolysis and glutamine metabolism in this study.

To explore the metabolomic profiles of erlotinib-resistant NSCLC cells and understand the metabolic adaptation of the cells, we performed ^1^H 1D NMR experiments to analyze metabolites changes in these cells. Results from metabolic profiling of HCC827 and HCC827ER cells identified the metabolites responsible for the different phenotypes between HCC827 and HCC827ER cells (Figure 6). The glutamine and glycolysis metabolism were significantly up-regulated in HCC827ER cells compared to HCC827 cells. The concentration of metabolites including glutamine (Figure 6A, **P < 0.01), glutamate (Figure 6B, *P < 0.05), glucose (Figure 6C, *P < 0.05), and lactate (Figure 6D, *P < 0.05) decreased when treated with compound 968 combined with erlotinib in HCC827ER cells. It is interesting that HCC827ER cells stored abundant adenosine triphosphate (ATP) for cell metabolism (Figure 6E, ***P < 0.001), and the concentration of ATP decreased greatly from 29.8 μM to 4.1 μM (**P < 0.01) when HCC827ER cells were treated with compound 968 combined with erlotinib. Total 64 metabolites detected by ^1^H NMR analysis were presented in Table S1. A simple pathway map of metabolites from glycolysis and glutamine metabolism was showed in Figure 6F. These results indicate clearly that the glycolysis and glutamine metabolism are enhanced in HCC827ER cells compared with HCC827 cells. Taken together, we can conclude that compound 968 reverses acquired erlotinib resistance in non-small cell lung cancer by inhibiting not only glutamine metabolism but also glycolysis.

## DISCUSSION

Epidermal growth factor receptor (EGFR), a kind of tyrosine kinase receptors, plays a critical role in regulating multiple cellular processes, including proliferation, survival and apoptosis. It is a member of the HER family, which also includes HER2 (ErbB2), HER3 (ErbB3), HER4 (ErbB4) [32, 33]. The constitutive activation of EGFR signaling pathway, caused by gene mutations or by gene amplification or both, has been demonstrated to have close connection with the initiation, progression and poor prognosis of NSCLC [34]. Therefore, EGFR becomes one of the landmark targets for NSCLC therapy. However, almost all the patients with initial dramatic responses to gefitinib or erlotinib ultimately become resistant to them mostly within 6–12 months, which has been defined as “acquired resistance” [12]. Thus, there is an urgent need to develop effective strategies to overcome the resistance.

In the present study, we explored a new way to overcome acquired erlotinib resistance in NSCLC by inhibiting the glutamine metabolism. The enhanced glutamine metabolism in cancer cells has already been exploited for cancer diagnosis and treatment. Because of this, the enzyme GLS has become a key target for small molecule therapeutic intervention in recent years [35]. Several glutaminase inhibitors were designed and synthesized for targeting glutamine metabolism in cancer therapy. However, because toxicity, side-effects, they still have not used in cancer treatment [34–36]. In our previous work, when we studied Rho GTPases regulated cellular transformation, we found a novel glutaminase inhibitor-compound 968, it inhibited potently the growth of breast cancer cell, however, no effects on the growth and morphology of normal human mammary epithelial cells and 3T3 cells [23]. To check whether compound 968 has side effects on lung cell lines, we used primary human bronchial epithelial (HBE) cells to do experiments described in Supplementary Methods. The results demonstrated that compound 968 did not influence the cell proliferation, apoptosis and cell cycle of HBE cells even at the concentration of 40 μM (Figure S3), suggesting that compound 968 also has low or no toxic effects on normal lung cells.

Recently, researchers found that glutamine uptake and GAC expression is markedly enhanced not only in NSCLC cells sensitive to erlotinib, but also in cells resistant to erlotinib [21, 22]. Similar results were observed in our study, we found that GAC expression was much higher in HCC827ER cells than that in HCC827 cells. We also found that compound 968 inhibited the cell growth of erlotinib-resistant NSCLC cell line HCC827ER, suggesting compound 968 re-sensitized HCC827ER cells to erlotinib *in vitro*. To figure out the molecular mechanism for compound 968 re-sensitizing HCC827ER cells to erlotinib, we checked EGFR expression levels for different treatments. Although erlotinib or compound 968 treatment alone had no or weak effect on the expression of EGFR, the combination of compound 968 and erlotinib decreased the expression of EGFR obviously. This can explain why combination of compound 968 and erlotinib has synergized inhibitory effects on the growth of HCC827ER cells because they inhibited not only GAC but also EGFR.

Mutations that substitute methionine for threonine at position 790 (T790M) in the EGFR kinase domain seems to occur in approximately 50% of lung adenocarcinomas from patients with acquired resistance to the EGFR-TKIs [13, 36, 37]. Therefore, the T790M mutation in EGFR is regarded as a marker for acquired resistance to EGFR-TKIs. Another mechanism by which patients develop acquired resistance to EGFR inhibitor occurs via the amplification of the *MET* oncogene [38, 13]. Approximately 5–20% of the NSCLC patients undergo amplification of the *MET* after long period under the EGFR-TKI treatment [39]. In 2010, Kenichi Suda et al. reported that HCG827ER, a cell line with acquired resistance to erlotinib derived from HCC827, had *MET* amplification without EGFR T790M mutation [40]. Therefore, targeting T790M mutant and *MET* amplification may be a potential strategy to overcome acquired resistance of EGFR-TKIs. In our experiments, several typical human NSCLC cell lines associated with EGFR mutant were used to investigate the cell growth inhibition by compound 968 and erlotinib. Compound 968 combined with erlotinib significantly decreased cell proliferation of HCC827ER cells harboring *MET* amplification and NCI-H1975 cells with T790M mutant. It also moderately suppressed the growth of A549 cells with EGFR wild type. However, the combination of compound 968 and erlotinib did not block the cell proliferation of NCI-H1650 cells with PTEN mutant. These results indicate that the combination of compound 968 and erlotinib can reverse acquired erlotinib resistance in NSCLC with EGFR (T790M) mutant and *MET* amplification. This combination should be a promising therapeutic strategy for treatment of acquired resistance to EGFR-TKIs in NSCLC.

The metabolic characteristics of cancer cells were distinguished from that of normal cells, and the identification of metabolic profiles relevant to erlotinib-resistant HCC827ER cells was helpful to understand the metabolic adaptation of the cells. The glutamine and glycolysis metabolism was enhanced in HCC827ER cells compared to that in HCC827 cells, which was consistent with previous reports [21]. It was interesting that compound 968 combined with erlotinib suppressed not only glutamine metabolism, but also glycolysis. These results indicate that the combination of compound 968 and erlotinib inhibits the growth of erlotinib-resistant NSCLC cells by suppressing whole cellular metabolism, not just glutamine pathway alone.

All these results indicate that both EGFR and GAC play crucial roles in regulating cancer cell function. Treatment with EGFR inhibitors, such as erlotinib and gefitinib, may cause several types of side effects, including skin rash, acute lung disease, and diarrhea [44, 45]. The combination of an EGFR-TKI and a GAC inhibitor may optimize both antitumor efficacy and safety, and provide a new therapeutic strategy for the EGFR-TKI resistant NSCLC.

## MATERIALS AND METHODS

### Reagents

Compound 968 was purchased from Calbiochem (Merck Millipore, Darmstadt, Germany). Erlotinib hydrochloride was obtained from Biovision Inc. Compound 968, erlotinib, gefitinib, and cisplatin were dissolved in DMSO, and used at the indicated concentrations. Antibodies against β-actin and secondary antibodies were purchased from Cell Signaling Technology (Danvers, MA, USA). Mouse monoclonal EGFR antibody was obtained from Becton, Dickinson and Company. Rabbit polyclonal GAC antibody was made in a company. RPMI 1640 Medium (with 300 mg/L L-Glutamine) and Advanced RPMI 1640 Medium (without L-Glutamine) were purchased from GIBICO (Carlsbad, CA, USA). Crystal violet and other analytical grade chemicals were purchased from Sigma-Aldrich (St. Louis, MO, USA).

### Cell culture

Human NSCLC cell lines HCC827 (*EGFR* exon 19 deletion [delE746-A750]) and erlotinib-resistant HCC827ER cells harboring *MET* gene amplification were kindly provided by Professor Shi-Yong Sun (Department of Hematology and Medical Oncology; Emory University School of Medicine and Winship Cancer Institute, USA). HCC827ER cells were developed by chronic exposure HCC827 cells to erlotinib at increasing concentrations as described previously [40]. Wild type (A549) and mutated [NCI-H1975 (L858R, T790M), NCI-H1650 (phosphatase and tensin homologue loss)] EGFR-expressing cell lines were obtained from the American Type Culture Collection (ATCC). All cell lines were cultured in RPMI- 1640 medium, supplemented with 10% FBS at 37°C in a humidified 5% CO_2_ incubator.

### Cell proliferation assay

HCC827, HCC827ER, NCI-H1975, NCI-H1650, and A549 cells were seeded in 24-well plates at a density of 2,000 cells per well in 0.5 ml of media. For erlotinib resistance assay, cells were treated with series of concentrations of erlotinib (0.01, 0.05, 0.1, 0.5, 1, and 2 μM). For erlotinib and compound 968 combination assay, cells were treated with compound 968, or erlotinib, or compound 968 combined with erlotinib, respectively. After 48 h incubation, cells were fixed in 10% formalin and stained with 0.1% crystal violet. Dye was extracted with 10% acetic acid and the relative proliferation was determined at 595 nm.

### Soft agar assay

HCC827 and HCC827ER cells were trypsinized and counted, 1 × 10^4^ cells were resuspended in 0.3% agar medium containing 10% FBS with erlotinib (1 μM) or DMSO. The mixture was plated on top of a solidified layer of RPMI-1640 supplemented with 0.5% agarose and 10% FBS. Cells were fed weekly by adding 1 ml of 0.3% agar medium containing 10% FBS with erlotinib (1 μM) or DMSO. After 2 weeks, colonies larger than 50 μm were scored and the cell growth in soft agar was calculated.

### Growth in glutamine free medium

HCC827 and HCC827ER cells were seeded into 12- well culture plates (1 × 10^5^ cells per well) and allowed to adhere to the wells overnight. Media were changed with advanced RPMI 1640 medium without L-Glutamine (300 mg/L). Cells were cultured from day 1 to day 6, and the cell growth was determined by cell proliferation assay.

### Knockdown and transfection

For knockdown experiments, the cells were seeded the day before siRNA transfection to reach 50% confluency at the time of transfection. Three distinct Stealth siRNAs of GAC (Invitrogen, Cat.HSS104192; HSS104193; HSS178458) were used as described previous [28]. A nonspecific oligonucleotide was used as a negative control and the knockdown efficiency was determined by Western blot. Cell proliferation after GAC knockdown was determined by crystal violet staining at day 2, 4, and 6. For plasmid-based transfections, cells transfected with GAC siRNA for 24 h were seeded the day before to reach 80% confluency at the day of transfection. Then, cells were transfected with 2 μg pCDNA 3.1-V5-GLS [28]. The rescue efficiency was determined by Western blot and cell proliferation was determined by crystal violet staining at day 2, 4, and 6.

### Saturation density assay

Saturation density assay was conducted as previously described [46]. Briefly, cells with 70% to 80% confluence were trypsinized and plated into 12-well culture plates (1 × 10^5^ cells per well) in 1 mL of media. Cells were allowed to adhere to the wells overnight and then counted (day 0). Media were changed every 2 days by fresh media containing compound 968(0, 5, 10, 20, and 40 μM). Cells were counted at the 6 days by a hemocytometer.

### Glutaminase activity assay

Glutaminase activity was determined as previously described [28, 47] with some modification. Isolated mitochondria were incubated with 57 mM Tris-acetate (pH 8.6) and 0.225 mM EDTA by rotating at 37°C, in a final volume of 110 μL and a final concentration of 17 mM. The reaction proceeded for 1 hr and stopped by adding 10 μL of ice-cold 3M hydrogen chloride (HCl). An aliquot of the quenched reaction mixture (10 μL) was added to an incubation containing 114 mM Tris-HCl (pH 9.4), 0.35 mM adenosine diphosphate (ADP), 1.7 mM nicotinamide adenine dinucleotide (NAD), and 6.3 U/ml glutamate dehydrogenase to give a final volume of 446 ml. The reaction mixture was incubated at room temperature for 45 min. The absorbance of each sample was read at a wavelength of 340 nm and the glutaminase activity was calculated.

### Western blot analysis

Cells were lysed in lysis buffer containing phosphatase inhibitor cocktail and proteinase inhibitor cocktail (Sigma), and the protein concentrations were determined by the BCA Protein Assay Kit (Pierce Biotechnology). Total protein (20–40 mg) was subjected to SDS-PAGE and transferred to PVDF membranes (Milipore). The membranes were blocked with 5% skim milk and incubated with antibodies to β-actin, EGFR, and GAC. After washing 3 times, the membranes were incubated for 1 hour at room temperature with horseradish peroxidase-conjugated secondary antibodies. Proteins were visualized by using an enhanced chemiluminescence detection kit (ECL; Amersham Biosciences UK Ltd, Little Chalfont, UK). Band intensities were quantified densitometrically using ImageJ software.

### Metabolic analysis

Metabolomic profiles were obtained to assess the relative distribution of various cellular metabolites of HCC827 and HCC827ER cells. Cells were collected and quickly frozen. Further sample preparation, metabolic profiling, peak identification and curation were performed by Anachro Technologies Inc. (Wuhan, China) using their described methods [48, 49]. All NMR experiments were carried out on an Agilent DD2 600 MHz spectrometer (^1^H resonance frequency) equipped with a triple-resonance cryoprobe. Identification of the metabolites in the cells was carried out from the 1D NMR data using the software Chenomx NMR Suite 8.0 (Chenomx Inc., Edmonton, Canada).

### Statistical analysis

Results are represented by mean ± S.D. Metabolite concentrations were exported from the Chenomx software and prinicipal component analysis (PCA) was conducted on all data sets initially to evaluate overall patterns and check for outliers. Statistical significance was tested by one-way ANOVA, with *P*-value of less than 0.05 considered statistically significant. All statistical analyses were conducted using GraphPad Prism 5.0.

## SUPPLEMENTARY MATERIALS FIGURES, TABLE AND METHODS


