# Supplementary material for: Inhibition of mitochondrial glutaminase activity reverses acquired erlotinib resistance in non-small cell lung cancer
Source: Oncotarget. 2015 Nov 13;7(1):610–21. doi: 10.18632/oncotarget.6311 (PMC4808021; doi:10.18632/oncotarget.6311)
Supplement: Supplementary file 1 [file oncotarget-07-0610-s001.pdf]

Inhibition of mitochondrial glutaminase activity reverses acquired erlotinib resistance in non-small cell lung cancer

Supplementary Materials

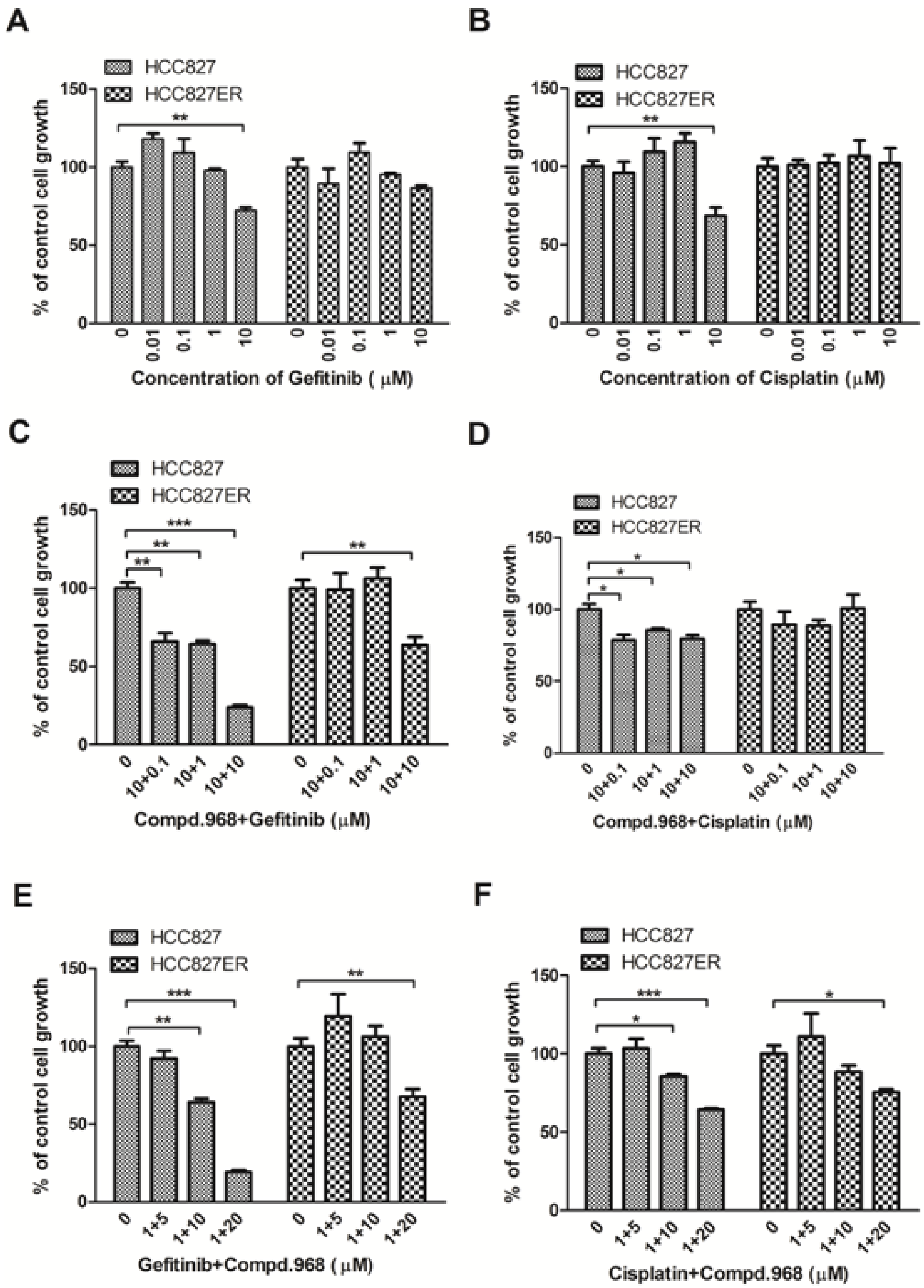

Supplementary Figure S1: (A and B) Cell growth of HCC827 and HCC827ER cells treated with gefitinib (0, 0.01, 0.1, 1, and 10 μM) or cisplatin (0, 0.01, 0.1, 1, 10 μM) respectively for 48 hours. (**C** and **D**) Cell growth of HCC827 and HCC827ER cells treated with compound 968 (10 μM) combined with increasing concentrations of gefitinib (0, 0.1, 1, and 10 μM) or with increasing concentrations of cisplatin (0, 0.1, 1, and 10 μM) respectively for 48 hours. (**E** and **F**) Cell growth of HCC827 and HCC827ER cells treated with gefitinib (1 μM) or cisplatin (1 μM) combined with increasing concentration of compound 968 (0, 5, 10, and 20 μM).

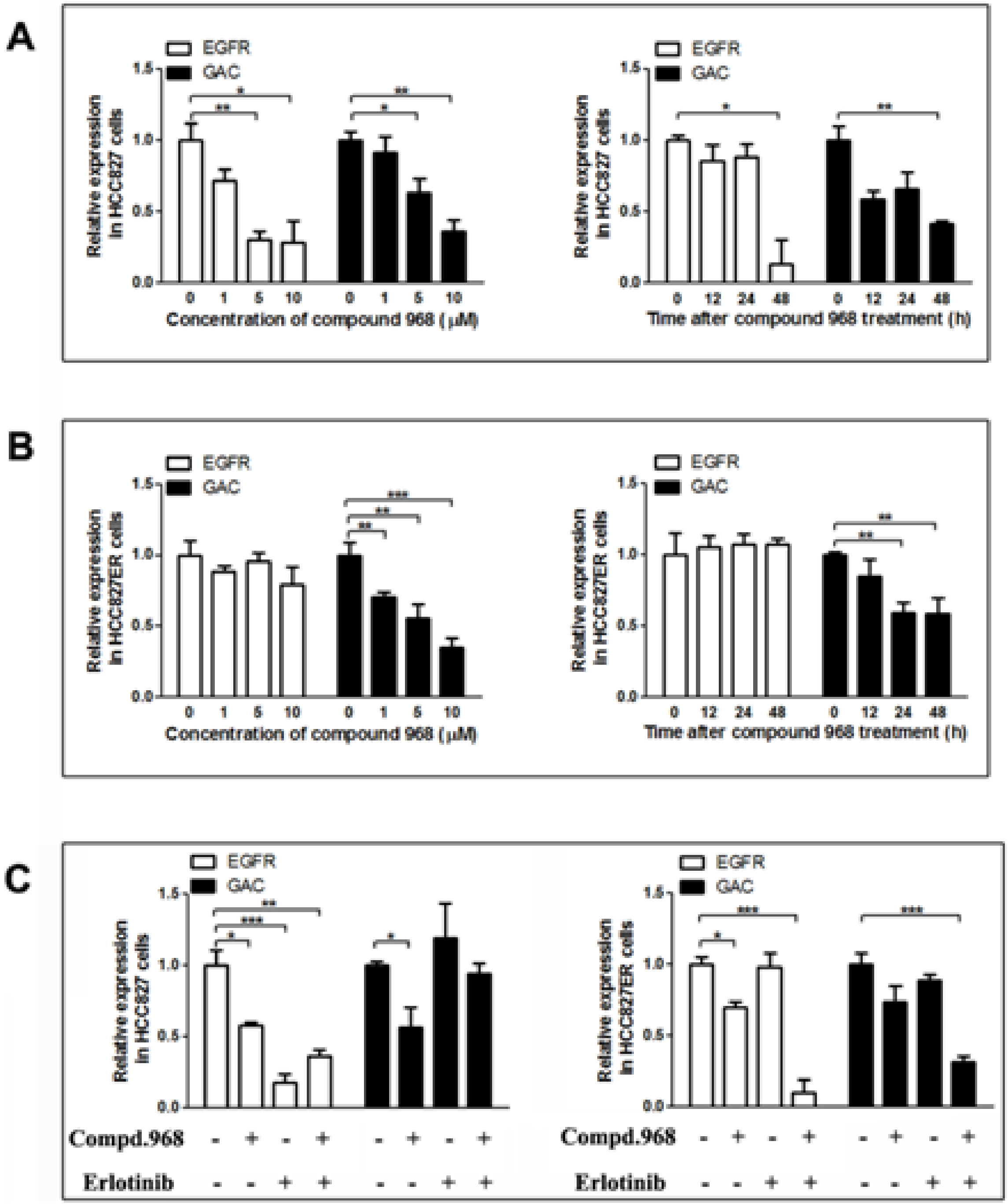

Supplementary Figure S2: (A) Statistical analysis of band intensities of Figure 5A.The proteins expressions of EGFR and GAC in HCC827 cells treated with compound 968 at different concentrations (left panel) or for different times (right panel). (**B**) Statistical analysis of band intensities of Figure 5B. The proteins expressions of EGFR and GAC in HCC827ER cells treated with compound 968 at different concentrations (left panel) or for different times (right panel). (**C**) Statistical analysis of band intensities of Figure 5C. The proteins expressions of EGFR and GAC in HCC827 cells (left panel) and HCC827ER cells (right panel) treated with compound 968 (10 μM) combined with erlotinib (1 μM). Data are shown as means ± S.D. of three experiments. $^{*}P < 0.05$, $^{**}P < 0.01$, and $^{***}P < 0.001$.

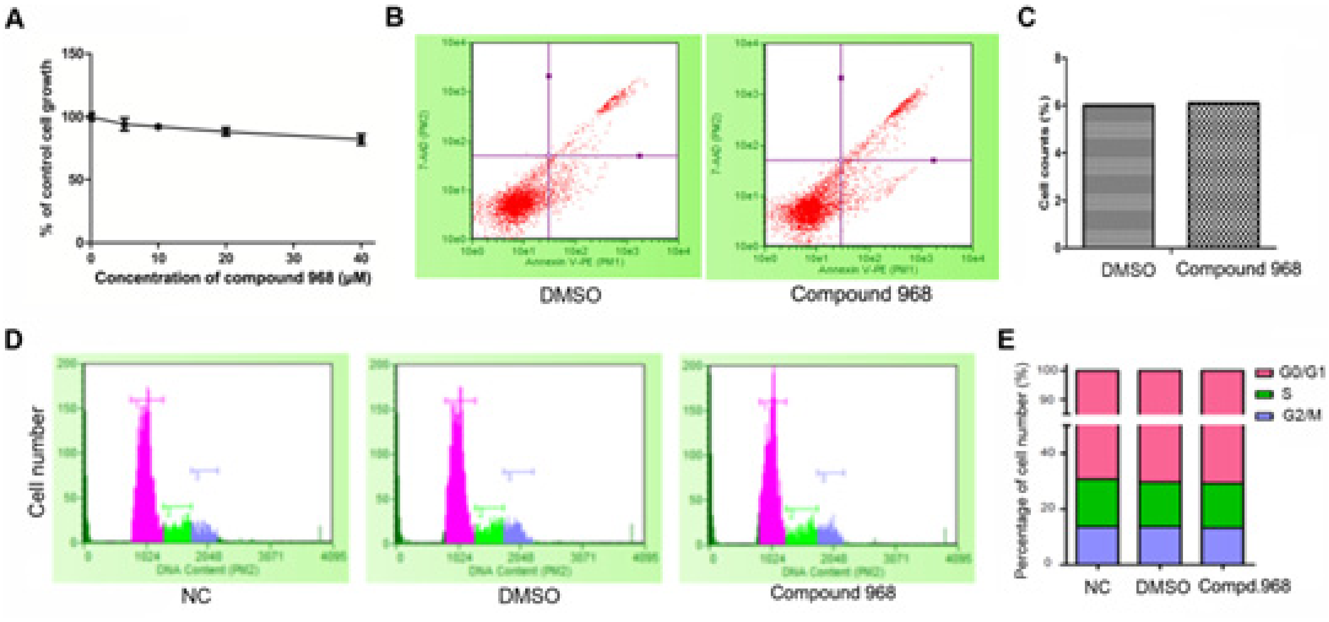

Supplementary Figure S3: (A) Cell proliferation of HBE cells treated with compound 968 (0, 5, 10, 20, and 40 μM) for 48 hours. (**B**) Cell apoptosis of HBE cells treated with compound 968 (40 μM) for 48 hours. (**C**) Statistical analysis of the cell apoptosis assay. (**D**) Cell cycle analysis of HBE cells treated with compound 968 (40 μM) for 48 hours. (**E**) Statistical analysis of cell cycle assay results.

Supplementary Table S1: Concentrations of metabolites detected in each cell line

Metabolites	Concentration (μM)		
	HCC827	HCC827ER	HCC827ER treated with compd.968+erlotinib
**Amino acids and the derivatives**			
4-Aminobutyrate	7.7	14.4	21.2
Alanine	297.1	300.0	324.9
Arginine	0.7	67.9	36.9
Asparagine	85.4	130.1	110.4
Aspartate	112.3	82.6	143.8
Betaine	18.3	8.7	13.3
Citrulline	142.0	37.5	119.9
Creatine	281.5	300.7	239.5
Cysteine	190.5	126.4	88.1
Glutamate	843.1	1234.6	999.8
Glutamine	63.1	105.9	73.8
Glycine	630.6	254.7	518.8
Isoleucine	88.0	81.1	94.6
Leucine	153.1	169.2	157.3
Lysine	130.2	101.1	144.2
Methionine	47.1	49.9	57.8
Phenylalanine	70.9	74.9	73.2
Proline	273.9	209.6	289.9
Pyroglutamate	63.6	75.7	92.4
Sarcosine	0.9	4.1	2.5
Serine	178.4	167.9	157.6
Taurine	517.9	665.5	865.5
Threonine	82.4	88.5	119.7
Tryptophan	16.9	15.5	19.9
Tyrosine	87.6	75.5	91.4
Valine	119.2	113.6	123.8
β-Alanine	22.8	49.0	36.4
**Organic acids**			
3-Hydroxyisovalerate	1.3	0.6	0.8
3-Methyladipate	4.5	1.6	3.3
Acetate	334.8	45.7	265.9
Formate	33.7	7.3	34.9

Fumarate	9.5	11.0	13.3
Lactate	2218.3	5293.5	2782.9
Malate	61.3	108.2	75.2
Malonate	4.7	19.2	10.3
Succinate	28.3	4.4	38.9
**Sugars**			
Glucose	0.9	35.5	8.1
Glucose-1-phosphate	0.3	12.3	0.9
Lactose	28.1	60.7	35.0
UDP-N-Acetylglucosamine	20.7	40.3	107.3
**Alcohols**			
Ethanol	20.4	15.0	22.2
Methanol	15.4	2.9	16.0
myo-Inositol	298.9	182.7	219.7
**Nucleic acid components**			
Adenine	12.7	12.0	13.1
ATP	1.5	29.8	4.1
Guanosine	35.4	62.9	33.5
Hypoxanthine	299.8	73.6	254.0
Inosine	68.0	220.3	71.1
NAD+	0.8	6.6	2.4
Thymine	11.7	0.8	1.1
UDP-glucose	10.7	1.3	52.7
UDP-glucuronate	14.0	44.1	23.0
Uracil	275.6	92.3	219.7
Uridine	19.1	71.1	16.5
**Others**			
N-Acetylaspartate	7.5	38.3	43.7
Nicotinurate	78.6	35.6	74.4
Creatinine	1.0	13.7	8.9
Putrescine	53.3	78.8	80.3
Choline	352.2	159.2	192.9
O-Phosphocholine	137.6	553.2	258.4
3-Methylxanthine	0.9	21.1	2.1
Caffeine	4.2	0.6	1.6
1-Methylnicotinamide	17.0	18.0	17.5
Glutathione	21.7	354.8	104.4
Pantothenate	4.4	1.5	3.2

SUPPLEMENTARY METHODS

Cell culture

Primary human bronchial epithelial (HBE) cells were purchased from ScienCell Research Laboratories (Catalog #3210, San Diego, CA, USA) and maintained in bronchial epithelial cell medium (ScienCell, BEpiCM, 3211). HBE cells were incubated at 37°C in a humidified 5% CO_2 atmosphere and cultured for no more than three passages before analysis.

Flow cytometry assay

The percentage of apoptotic cells was determined by double staining with Annexin V- Phycoerythrin (PE) and 7-aminoactinomycin D (7-AAD). HBE cells were treated with fresh RPMI 1640 medium or medium containing compound 968 at the concentration of 20 μM for 48 h. Cells were harvested, fixed, and double-labeled with Annexin V-PE and 7-AAD (KeyGEN Biotech, Nanjing, China). For cell cycle analysis, HBE cells were treated with compound 968 at the concentration of 20 μM for 48 h. Cells were suspended in phosphate-buffered saline solution containing 40 μg/mL propidium iodide, 0.02% Triton X-100, and 50 μg/mL ribonuclease A. Samples were incubated in the dark at room temperature for 30 min and stored at 4°C until analysis. Flow cytometric analysis of stained cells was done on the Becton Dickinson FACS Calibur (Becton Dickinson).

Gefitinib and cisplatin sensitization evaluation

HCC827 and HCC827ER cells were treated with gefitinib (0, 0.01, 0.1, 1, 10 μM) or cisplatin (0, 0.01, 0.1, 1, 10 μM), or compound 968 combined with gefitinib, or compound 968 combined with cisplatin. After 48h incubation, cells were fixed in 10% formalin and stained with 0.1% crystal violet. Dye was extracted with 10% acetic acid and the relative proliferation was determined at 595 nm.
